# The contributions of ammonia oxidizing bacteria and archaea to nitrification-dependent N_2_O emission in alkaline and neutral purple soils

**DOI:** 10.1038/s41598-022-23084-1

**Published:** 2022-11-19

**Authors:** Lei Hu, Zhixin Dong, Zheng Wang, Liwei Xiao, Bo Zhu

**Affiliations:** 1 Key Laboratory of Mountain Surface Processes and Ecological Regulation, Institute of Mountain Hazards and Environment, Chinese Academy of Sciences, #9, Block 4, Renminnanlu Road, Chengdu, 610041 Sichuan China; 2 University of Chinese Academy of Sciences, Beijing, 100049 China; 3 Institute of Geographic Sciences and Natural Resources Research, Chinese Academy of Sciences, Beijing, 100101 China; 4 Chengdu University of Technology, Chengdu, 610059 China

**Keywords:** Biochemistry, Biogeochemistry, Environmental sciences

## Abstract

Nitrification is believed to be one of the primary processes of N_2_O emission in the agroecological system, which is controlled by soil microbes and mainly regulated by soil pH, oxygen content and NH_4_^+^ availability. Previous studies have proved that the relative contributions of ammonia oxidizing bacteria (AOB) and archaea (AOA) to N_2_O production were varied with soil pH, however, there is still no consensus on the regulating mechanism of nitrification-derived N_2_O production by soil pH. In this study, 1-octyne (a selective inhibitor of AOB) and acetylene (an inhibitor of AOB and AOA) were used in a microcosm incubation experiment to differentiate the relative contribution of AOA and AOB to N_2_O emissions in a neutral (pH = 6.75) and an alkaline (pH = 8.35) soils. We found that the amendment of ammonium (NH_4_^+^) observably stimulated the production of both AOA and AOB-related N_2_O and increased the ammonia monooxygenase (AMO) gene abundances of AOA and AOB in the two test soils. Among which, AOB dominated the process of ammonia oxidation in the alkaline soil, contributing 70.8% of N_2_O production derived from nitrification. By contrast, the contribution of AOA and AOB accounted for about one-third of nitrification-related N_2_O in acidic soil, respectively. The results indicated that pH was a key factor to change abundance and activity of AOA and AOB, which led to the differentiation of derivation of N_2_O production in purple soils. We speculate that both NH_4_^+^ content and soil pH mediated specialization of ammonia-oxidizing microorganisms together; and both specialization results and N_2_O yield led to the different N_2_O emission characteristics in purple soils. These results may help inform the development of N_2_O reduction strategies in the future.

## Introduction

N_2_O is a trace greenhouse gas with 265 times the warming potential of CO_2_ (on a 100-year scale) in the atmosphere and acts to deplete stratospheric ozone^1^. The global concentration of N_2_O in the atmosphere was 328.8 ppb in 2015 with a 21% increase since the industrial revolution^2^. The sustained emissions at the current rate will result to increase by another 18% until 2030 from current projections indicate^3^. Fertilized agricultural soil is a hot spot for emissions substantial N_2_O to atmosphere, accounting for about 60% of global atmospheric N_2_O emissions^4^. However, increasing demand for food, animal husbandry and biomass energy will inevitably accompany the continuous application of chemical fertilizers. Therefore, it is particularly important to understand the mechanism responsible for N_2_O production from fertilized soil to find optimal measures for regulating N_2_O emissions for sustainable agriculture.

In agro-ecosystems, N_2_O is produced by nitrification and denitrification driven by soil microorganisms^5,6^, and some abiotic processes also contribute a small part of it^7^. Nitrification is a main N_2_O production process under a suitable soil oxygen content. Ammonia oxidation (i.e. NH_3_ being oxidized to NO_2_^−^, via the intermediate product NH_2_OH) is considered to be the primary and rate-limiting step of nitrification. Both ammonia-oxidizing archaea (AOA) and ammonia-oxidizing bacteria (AOB) have the genetic potential for ammonia oxidation which cause production of N_2_O^8,9^. AOB could produce N_2_O which acts as the intermediate by the incomplete NH_2_OH oxidation to NO^10^, or by regulating nitrifier denitrification of reduction NO_2_^−^ to NO and N_2_O^11^. In contrast, N_2_O produced by AOA attributes to multiple processes conventionally detected in pure and enrichment cultures, but there was no clear evidence to date for supporting production of N_2_O by AOA enzymatic catalytic reaction in soils^12^. In addition, the newly discovered and widespread complete ammonia oxidizers (comammox) which act as one of ammonia-oxidizing functional guilds constitute remains uncertainty of influence on N_2_O emission, though some studies suggested it playing a minor role than AOB^13,14^.

Nitrification inhibitors have been widely applied in agricultural soils to reduce transformation of NH_4_^+^-N to NO_3_^–^N, thus improving N use efficiency^15^. One of the key functional enzymes in both AOA and AOB ammonia oxidizers is ammonia monooxygenase (AMO) which directly catalyzed the process of N_2_O synthesis. Acetylene (C_2_H_2_) is a non-selective nitrification inhibitor for AMO, thus inhibiting ammonia oxidation of both AOA and AOB at a low concentration (0.1–10 Pa)^16,17^. Meanwhile, 1-octyne is a selective inhibitor that can inhibit AOB activity but not AOA in soils, and it can be used to distinguish the relative contribution of AOB and AOA to nitrification^18–21^.

Substantial studies have found that many factors including soil types and environmental factors determine the abundance, activities and relative contribution to N_2_O emission of AOA and AOB, especially the soil pH and inorganic nitrogen (N) supply^22–24^. For example, the abundance and activity of AOB increased in ammonium concentration-rich soils, whereas AOA act as not affected or inhibited^16,18^. In unfertilized or acidic soils, abundance and activity of AOA are much higher than those of AOB^25,26^. Wang et al.^21^ reported that nitrogen fertiliser-induced N_2_O emissions are attributed 70.5 ~ 78.1% by AOB and 18.7 ~ 19.7% by AOA using the method of inhibitors both in acidic (pH = 6) and alkaline (pH = 8) arable soils of China. Similarly, using the method of inhibitors, Yang et al.^27^ found that AOB was the key microbial player in alkaline soil which contributing about 85% of nitrification-related N_2_O, while 78% of nitrification-related N_2_O was contributed by AOA in acidic soil. In addition, the relative contribution of AOB and AOA to N_2_O emissions was also regulated by type and amount of applied synthetic Hink et al.^24^ found that high ammonia addition stimulated the production of N_2_O from AOB, but AOA dominance during low ammonium supply. However, Fu et al.^28^ illustrated that the relative contribution of AOB to N_2_O emissions in the treatment of no N applied was larger than the treatment of ammonium-N addition in both acidic (pH = 5.5) and alkaline (pH = 7.9) soils and treatment of urea-N addition in alkaline soil.

Numerous studies have investigated the relative contribution and influence factors of AOA and AOB to N_2_O emission, but no consensus exists regarding the mechanisms for explaining the diversities because of complex mechanism of abundance change and N_2_O yields of AOA and AOB accompanied by spatial and temporal heterogeneity of environmental conditions and soil properties. Owing to the considerable N_2_O emission source caused by the heavy N fertilizer application on farmland in purple soil in mountain area of the Upper Yangtze River watershed^29,30^, it is imperative to put forward specific measures to alleviate N_2_O emission for achieving the strategic goal of “carbon reduction” in this region. Therefore, we conducted a microcosm incubation experiment with two contrasting purple soils, using newly developed inhibitor 1-octyne and molecular biology method to assess the factors affecting N_2_O productions, yield and gene abundance associated with AOA and AOB in different soils. The objective is to acquire a more profound understanding of the mechanism of ammonia oxidation process and promote the development of low-emission technology in agriculture.

## Materials and methods

### Soil sampling

In Sep 2020, two test surface soil samples were collected from a long-term fertilization experiment plots (5 m × 1.5 m, triplicate plots of each test soil) at Yanting Agro-Ecological Station of Purple Soil, Chinese Academy of Sciences (N 31°16′, E 105°28′ ), located in the central Sichuan Basin, upper Yangtze River, China. The average temperature was 17.3 °C and the annual mean precipitation was 863 mm of which approximately 70% occurs from May to September at this site. The cropping system is summer maize-winter wheat rotation there and N–P_2_O_5_–K_2_O was applied at 150–90–36 kg ha^−1^ for maize and 130–90–36 kg ha^−1^ for wheat, respectively.

Two test soils including a neutral (pH = 6.75 and named as SX below) and an alkaline (pH = 8.35 and named as PL below) were formulated from the similar parental bedrock of purplish sandstone with different weathering degree and it is less than 50 years since the soils formation^31,32^. They are the predominant soil types in hilly areas of the Upper Yangtze River watershed where was an important grain-producing area in southwest China and feeding more than 10% of the Chinese population.

Two test surface soils (0–15 cm) were collected using a soil auger and triplicate cores, which were along the longitudinal center line of the plots with a two meters interval, were pooled and homogenized for each plot. All soils were sieved through a 2 mm sieve after removing plant roots and debris and then divided into two parts. One part was used to measure soil water content and basic physical and chemical properties, the remained part of the soil was stored at 4 °C until the incubation experiment. Some basic physical and chemical properties of the two soils were shown in Table 1.

**Table 1:** Some basic physical and chemical properties of tested soils.

Parameter	SX	PL
pH	6.75 ± 0.13b	8.37 ± 0.01a
Total N (g kg^−1^)	0.66 ± 0.02b	0.80 ± 0.03a
SOC (g kg^−1^)	5.70 ± 0.29a	5.80 ± 0.09a
C/N ratio	25.94 ± 1.27a	21.65 ± 0.99b
CEC (cmok kg^−1^)	8.55 ± 0.02a	8.22 ± 0.09a
BD (g cm^−3^)	1.17 ± 0.03a	1.14 ± 0.01a
Porosity (%)	53.65 ± 0.86a	53.89 ± 0.73a
Clay (%)	17.86 ± 0.68b	30.82 ± 0.31a
Silt (%)	53.10 ± 2.05a	51.76 ± 1,87a
Sand (%)	29.04 ± 1.61a	17.42 ± 2.13b

Data are mean ± standard error (*n* = 3); Different letters within the same row indicate significant differences among treatments at *p* < 0.05 level.

### Microcosm experiment

To distinguish the relative contribution of different ammonia oxidation processes to N_2_O emission, we employed acetylene and 1-octyne as selective inhibitors to block the interaction of the ammonia oxidizers (i.e. AOB and AOA). The incubation experiments were conducted in 250-ml serum bottles with a butyl rubber stopper containing 18 g of soils (dry weight). The fresh soils were pre-incubated at 25 °C for 7 days to stabilize soil microbial activities in 250-ml serum bottles. After pre-incubation, soil was adjusted to 60% WFPS following amendment with sterilized water only (control, no N addition) or inorganic nitrogen solution (100 mg NH_4_Cl-N or KNO_3_-N g^−1^ soil_dw_). Then, the bottles were covered with lids and some pumped out air have replaced with pre-prepared ammonia oxidizer inhibitors acetylene (Ace, 0.01%, v/v) or 1-octyne (Oct, 5 μM aqueous, following Taylor et al.^20^). In total, nine treatments with three replicates were conducted as follows:

1. N-free (no N and no inhibitors)
2. N-free + Ace (no N and 0.01% acetylene)
3. N-free + Oct (no N and 5 μM 1-octyne)
4. NH_4_^+^ (100 mg g^−1^ NH_4_Cl-N and no inhibitors)
5. NH_4_^+^  + Ace (100 mg g^−1^ NH_4_Cl-N and 0.01% acetylene)
6. NH_4_^+^  + Oct (100 mg g^−1^ NH_4_Cl-N and 5 μM 1-octyne)
7. NO_3_^−^ (100 mg g^−1^ KNO_3_-N and no inhibitors)
8. NO_3_^−^ + Ace (100 mg g^−1^ KNO_3_-N and 0.01% acetylene)
9. NO_3_^−^ + Oct (100 mg g^−1^ KNO_3_-N and 5 μM 1-octyne)

All treatments conducted at 25 °C for 21 days. During this period, oxic conditions were maintained by aerated every 2 days and re-establishing the inhibition environment by addition acetylene (0.01% v/v) and 1-octyne (5 μM aqueous). The N_2_O emission from soils without inhibitors was contributed by nitrification (including contributions of AOB and AOA), denitrification and abiotic processes. Acetylene could inhibit ammonia oxidation both of AOA and AOB, so the N_2_O emission from AOA plus AOB was calculated by subtracting N_2_O emission in the “NH_4_^+^  + Ace” (“NO_3_^−^ + Ace” or “Ace”) treatment from values measured in the “NH_4_^+^” (“NO_3_^−^ ” or “N-free”) treatment. Because of 1-Octyne specifically inhibits AOB growth only, the N_2_O emission from AOA was calculated by subtracting N_2_O emission in the “NH_4_^+^ + Ace” (“NO_3_^−^ + Ace” or “Ace”) treatment from values measured in the “NH_4_^+^ + Oct” (“NO_3_^−^ + Oct” or “Oct”) treatment. N_2_O emission from AOB was calculated by subtracting AOA from values of AOA plus AOB.

### N_2_O and soil sampling

The 20 ml headspace gas samples were collected at 0, 1, 2, 3, 5, 7, 11, 14, 18 and 21 days by syringe (with a triple valve) during the whole incubation and N_2_O emission concentrate was determined with a gas chromatograph which equipped with a 63Ni electron capture detector for N_2_O concentrations (Agilent 7890B, USA). The gas measurement was calibrated using a known concentration of mixed gas (440 ppb N_2_O in mixed standard gas). The incubated soils were destructively sampled at 0, 7, 14 and 21 days. Soil samples were divided into two parts, a part stored at 4 °C for measuring soil contents of NH_4_^+^-N and NO_3_^−^-N; and another portion was kept at − 80 °C for DNA extraction. Soil contents of NH_4_^+^-N and NO_3_^–^N were extracted by 2 M KCl solution (soil: solution = 1:5 w/v), and then were filtered through 0.45 m filter membrane after shaking for 1 h. Extracts were analyzed by a continuous flow analyzer (Auto Analyzer 3, SEAL Analytical, Germany).

### DNA extraction and quantitive PCR (qPCR) analyses

The soil samples collected before incubation and after incubating 21 days were used to extract DNA because the fluxes of N_2_O emission have stabilized after incubating 21 days. According to the manufacturer's instructions, 0.5 g wet soil was used to extract DNA by using DNeasy PowerSoil DNA Isolation kit (QIAGEN, Germany). The length of extracted DNA was checked by 1% agarose gel electrophoresis and the concentration and qualification were measured by NanoDrop ND-1000 spectrophotometer (Nano Drop Technologies, Wilmington, DE, USA). And the ratio of A_260/280_ and A_260/230_ were in the range of 1.5–1.9 and 0.7–1.0, respectively. Purified DNA concentrations varied from 10.8 to 36.8 ng/μL. Soil DNA samples were stored at − 80 °C for quantitative PCR of *amoA* genes analyses.

AOB and AOA *amoA* genes of all treatments with three biological replicates were amplified and quantified using ABI 9700 real-time quantitative fluorescence PCR (Applied Biosystem, America); The sequence of AOB *amoA* amplified primers were *amoA*-1F (5'-GGGGTTTCTACTGGTGGT-3')/*amoA*-2R (5'-CCCCTCKGSAAAGCCTTCTTC-3')^33^ while ArchamoAF (5'-TAATGGTCTGGCTTAGACG-3')/ArchamoAR (5'-GCGGCCATCCATCTGTATGT-3')^34^ were used to amplify and quantify AOA *amoA* gene. Each 20-μl reaction system contained 10 μl GoTaq qPCR Master Mix (SYBR Premix Ex TaqTM), 0.5 μl of each primer (10 mM), 2 μl tenfold diluted DNA template and 7 μl sterilised pure water. The amplified reaction conditions of AOB and AOA were as follows: initial denaturation at 95 °C for 3 min, 40 cycles of denaturation at 95 °C for 30 s, annealing at 55 °C for 34 s and extension at 72 °C for 32 s, and extension at 72 °C for 5 min for data collection. The standard curves which were used to quantify the abundance of AOA and AOB *amoA* gene were obtained by ten-fold serial dilution of AOA and AOB plasmid DNA with known concentration (five points form 10^–3^ ~ 10^–7^ in this study). The melting curve analyses which were used to check the specificity of amplification products showed that amplification efficiencies of AOB and AOA *amoA* ranged from 92 to 98%, along with correlation coefficient (R^2^) of standard curves was 0.994 and 0.998 for AOB and AOA *amoA* genes, respectively.

### Calculations and statistical analysis

N_2_O fluxes were calculated using Eq. (1):

1 $${\text{F}} = \frac{{{\text{T}}_{0} }}{{{\text{T}} + {\text{T}}_{0} }} \times \frac{{\text{V}}}{{{\text{V}}_{0} }} \times \frac{{\text{M}}}{{\text{m}}} \times \frac{{{\text{dc}}}}{{{\text{dt}}}} \times 24 \times {\text{K}}$$

where F (ng N g^−1^ d^−1^) is the N_2_O emission rate; T_0_ (237 K) is the temperature at standard atmospheric state; T (°C) is the air temperature within the serum bottles; V (L) is the volume of the headspace; V_0_ (22.41 × 10^–3^ m^3^) is the molar volume at standard atmospheric state; M (28 g mol^−1^) is the molecular weight of N in N_2_O molecular; m (18 g) is the weight of oven-dried soil in the serum bottles; dc/dt is the change of N_2_O concentration (c) per unit interval (t); 24 is the number of hours in a day and K is the dimensional conversion coefficient.

The relative contributions of AOA and AOB to nitrification-driven N_2_O emission were calculated using Eqs. (2) ~ (4):

2 $${\text{N}}_{{2}} {\text{O}}\left( {{\text{AOA}}} \right)\left( \% \right) = \frac{{{\text{N}}_{2} {\text{O emission by AOA}}}}{{{\text{total of N}}_{2} {\text{O emission }}}}$$

3 $${\text{N}}_{{2}} {\text{O}}\left( {{\text{AOB}}} \right)\left( \% \right) = \frac{{{\text{N}}_{2} {\text{O emission by AOB}}}}{{{\text{total of N}}_{2} {\text{O emission}}}}$$

4 $${\text{N}}_{{2}} {\text{O}}\left( {{\text{Others}}} \right)\left( \% \right) = \frac{{{\text{N}}_{2} {\text{O emission by Others}}}}{{{\text{total of N}}_{2} {\text{O emission}}}}$$

where “total of N_2_O emission” was the cumulative N_2_O production of treatment without inhibitors addition after 21 days of incubation; “N_2_O emission by Others” was the cumulative N_2_O production of other processes after 21 days of incubation.

N_2_O yield for AOA, AOB and others was calculated using Eq. (5):

5 $${\text{N}}_{{2}} {\text{O}}\,\,{\text{yield}}_{{\left( {\text{x}} \right)}} = \frac{{{\text{N}}_{2} {\text{O emission}}_{{\left( {\text{x}} \right)}} }}{{{\text{NO}}_{3}^{ - } {\text{ produced}}}}$$

where x is AOA, AOB and others, “N_2_O emission_(x)_” and “NO_3_^−^ produced” are the cumulative N_2_O and nitrate over the whole 21 days for incubation, the unit of “N_2_O emission_(x)_” and “NO_3_^–^N produced” is mg N kg^−1^.

The statistical analysis was performed using SPSS 24.0 software (SPSS Inc., USA) and data in this study expressed as a mean ± standard error. Differences among different treatments were tested by ANOVA after Tukey's multiple range test by the least significant difference at the 5% level. Independent-samples t-test was performed for the statistical analysis of N_2_O yield between two soils. Pearson’s correlation between cumulative N_2_O emissions and AOB or AOA *amoA* gene copies were calculated. Figures were made using Origin 9.4 software (Origin Lab Corporation, Northampton, USA).

## Results

### Dynamics of soil mineral N concentration

**Figure 1:**
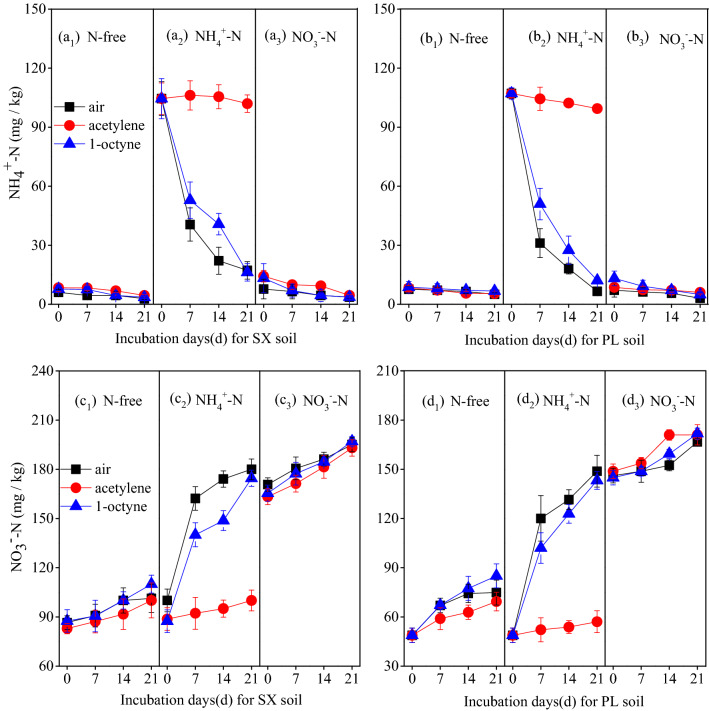
The dynamics NH_4_^+^-N content (**a**_**1**_ ~ **a**_**3**_, **b**_**1**_ ~ **b**_**3**_) and NO_3_^−^–N content (**c**_**1**_ ~ **c**_**3**_, **d**_**1**_ ~ **d**_**3**_) with different N fertilizers (N-free, ammonium-N, nitrate–N) in combination with air (no inhibitors), acetylene and 1-octyne during incubation of SX and PL soil, error bars represent standard errors of three biological replicates.

The exchangeable NH_4_^+^-N concentration of the “NH_4_^+^” treatment decreased rapidly from a value of 104 mg/kg to a value of 41 mg/kg and 107 mg/kg to 31 mg/kg in SX and PL soil at the first week, respectively. And the rate of decrease became slow in the following two weeks (Fig. 1a_2_, b_2_). Contrasting with the “NH_4_^+^” treatment, 1-octyne inhibited the decrease of exchangeable NH_4_^+^–N concentration effectively which showed a slower decrease rate in both of the two soils. As for “NH_4_^+^  + Ace” treatments, the conversion of NH_4_^+^ was completely inhibited, and no obvious decreasing trend was detected. Treatments without NH_4_^+^ addition maintained a low level of exchangeable NH_4_^+^-N concentration throughout the whole incubation, and there was no significant difference in these treatments whether inhibitors were applied or not (*P* > 0.05; Fig. 1a_1_, a_3_, b_1_, b_3_).

The exchangeable NO_3_^–^N concentrations correspondingly increased by the decreased of exchangeable NH_4_^+^-N concentrations in the “NH_4_^+^” treatments (Fig. 1c_2_, d_2_). As expected, 1-octyne present partly inhibited exchangeable NO_3_^–^N formation in the “NH_4_^+^  + Oct” treatments and acetylene almost completely inhibited the transformation of NH_4_^+^-N to NO_3_^–^N in the “NH_4_^+^  + Ace” treatments in the two soils. As for the treatments without NH_4_^+^ addition, there is no obvious difference regardless of the inhibitors were applied or not (*P* > 0.05; Fig. 1c_1_, c_3_, d_1_, d_3_).

### N_2_O emission fluxes and accumulative N_2_O emissions

**Figure 2:**
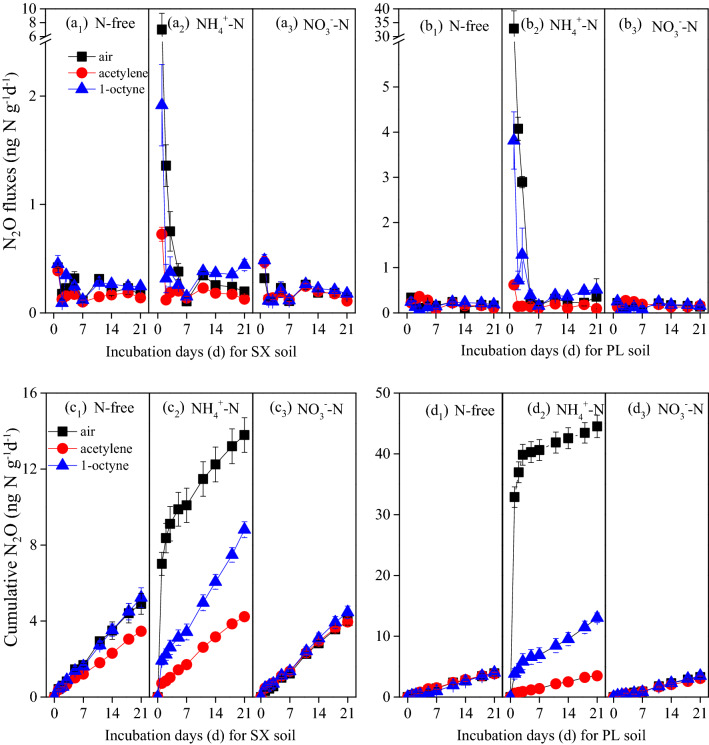
The N_2_O emission fluxes and accumulated fluxes with different N fertilizers (N-free, ammonium-N, nitrate–N) in combination with air (no inhibitors), acetylene and 1-octyne after 21 days incubation in SX (**a** and **c**) and PL (**b** and **d**) soil, error bars represent standard errors of three biological replicates.

The change trends of N_2_O fluxes varied with soil type. The treatments which addition NH_4_^+^ alone show a distinct N_2_O peak (7 and 33 ng N g^−1^d^−1^ for SX and PL experiment soil, respectively) at the first day of incubation, and quickly decrease at the following days (Fig. 2a_2_, b_2_). Acetylene addition has a significant effect in decreasing N_2_O production in the “NH_4_^+^  + Ace” treatment of the two soils throughout the whole incubation, indicating that AOA plus AOB contribute more N_2_O emissions than the abiotic and denitrification processes at aerobic and 60% WFPS experiment conditions. Relative to the acetylene addition, 1-octyne has a similar but slighter inhibition to N_2_O emissions in the “NH_4_^+^  + Oct” of the two soils, showing that different nitrification inhibitors had the selective inhibitory effects in alleviating N_2_O emissions as expected (Fig. 2a_2_, b_2_). Except for the NH_4_^+^ addition treatments, the results also reveal that treatments which with or without NO_3_^–^N show no significant difference in N_2_O emissions no matter the inhibitors present or not during the incubation period of 21 days (*P* > 0.05). And there is no emission peaks in these treatments without NH_4_^+^ addition and the fluxes show a periodic fluctuation until the end of incubation (Fig. 2a_1_, a_3_, b_1_, b_3_).

The accumulated N_2_O emissions varied with the soil type, types of nitrogen fertilizer and inhibitors (Fig. 2c_1_ ~ c_3_, d_1_ ~ d_3_). In the “NH_4_^+^” treatments, N_2_O accumulated emission which significantly greater than the rest treatments, reached 14 and 45 μg N kg^−1^ dry soil for SX and PL experiment soil at the end of incubation, respectively (*P* < 0.05; Table S1). When NH_4_^+^ addition was employed with 1-octyne, N_2_O accumulated emissions were dramatically decreased by 24.1% and 72.0% for SX and PL experiment soil, respectively, and the more intense inhibition by the acetylene addition which was reduced by 80.3% and 92.4% relative to the “NH_4_^+^” treatments. As for the NO_3_^−^ addition and no nitrogen fertilizer addition treatments, with or without an inhibitor, there was no significant difference for the two test soils at the end of incubation (*P* > 0.05; Table S1).

### The yields and relative contributions to N_2_O emission of AOB and AOA

**Figure 3:**
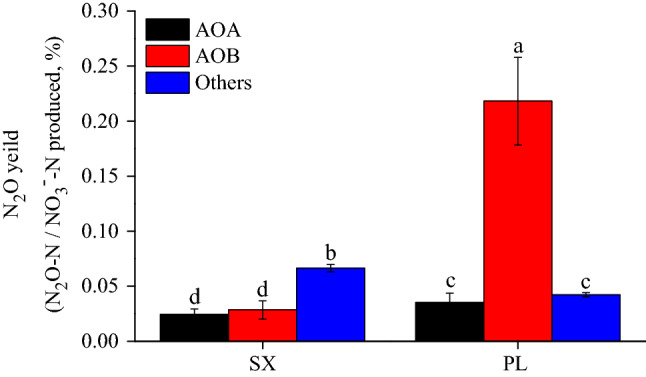
The yield of N_2_O associated with ammonia oxidation in NH_4_^+^-amended treatments; Different letters above the bars denote significant difference and the same letters denote no significant difference and error bars represent standard errors of three biological replicates.

The N_2_O yield (%) was defined as the rate of N_2_O production per unit nitrate content in soil over the whole 21 days for incubation which calculated by Eq. (5). The results showed that the yield of N_2_O in different ammonia oxidation processes changed with the soil type under the condition of adding NH_4_^+^ (Fig. 3). The N_2_O yield of AOB in PL soil was 0.22% which was significantly higher than that of SX (0.03%) (*P* < 0.01), and similar significant differences were found in the N_2_O yields induced by AOA (*P* < 0.05). However, for other processes (abiotic-induced or denitrification-induced), the N_2_O yield of SX soil was significantly higher than that of the PL soil (*P* < 0.05). Under the same soil conditions with NH_4_^+^ addition, the yield of N_2_O changed with different induction processes: in PL soils, the N_2_O yield induced by AOB was significantly higher than that induced by AOA and the other processes (*P* < 0.01); But in SX soils, the N_2_O yield induced by other processes was significantly higher than that induced by AOA and AOB (*P* < 0.01).

**Figure 4:**
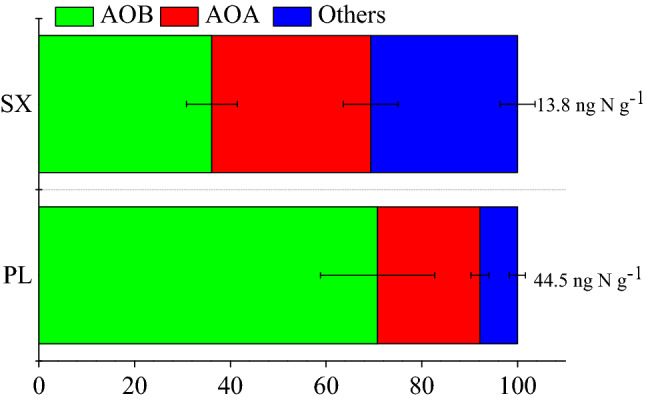
The relative contributions to N_2_O production of AOA and AOB from two soils with ammonia amendment treatments after 21 days incubation. Error bars represent standard errors of three biological replicates.

The relative contribution of AOA and AOB to N_2_O production varied significantly from the soil types with NH_4_^+^ amendment in the both of soils (Fig. 4). The fractions of N_2_O accumulate production of octyne-sensitive (AOB) were much higher than the octyne-resistent (AOA) in the PL soils. And for SX soil, the fraction of AOB production was slightly higher than the AOA and the others. In details, the fractions of N_2_O emission were 36.1%, 33.2% and 30.7% for AOB, AOA and others process in SX soil, respectively. The contributions of relevant AOB, AOA and others are 70.8%, 21.4% and 8.9% in PL soil, respectively.

### Abundance of AOA and AOB *amoA* genes

**Figure 5:**
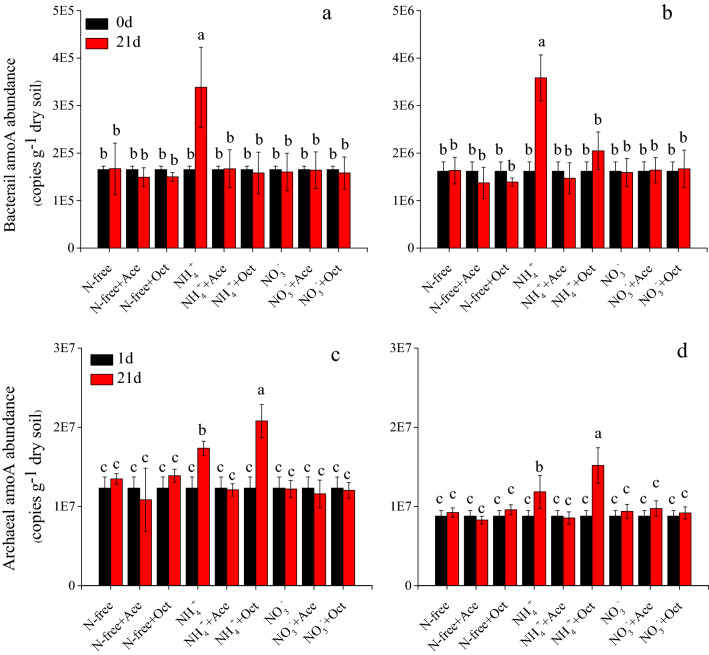
The AOB and AOA *amoA* genes abundance at 0 and 21 days of incubation in SX (**a** and **c**) and PL (**b** and **d**) soils; Different letters above the bars denote significant difference and the same letters denote no significant difference and error bars represent standard errors of three biological replicates.

At the start of incubation, the AOB *amoA* genes abundance were 1.65 × 10^5^ and 1.62 × 10^6^ copies g^−1^ dry soil in SX and PL soil, respectively (Fig. 5a, b) and different treatments have the equal abundance at the initiate of incubation. The NH_4_^+^ amendment significantly stimulated the increase of the AOB *amoA* genes, reaching at 3.38 × 10^5^ and 3.58 × 10^6^ copies g^−1^ in SX and PL soil at the day 21 of incubation, respectively (*P* < 0.01; Fig. 5a, b). There is no obvious difference of AOB *amoA* genes abundance among the other treatments throughout the incubation regardless of nitrogenous fertilizer and inhibitors were applied or not in both of the two soils.

As for AOA *amoA* genes, the AOA *amoA* genes abundance was 1.23 × 10^7^copies g^−1^ and 8.35 × 10^6^ copies g^−1^ dry soil in SX and PL soil at the initiate of incubation, respectively. When amendment with ammonia and 1-octyne, the abundance of AOA *amoA* genes increased observably than the other treatments in the two soils (*P* < 0.01; Fig. 5c, d). Presence of 1-octyne which was selected as inhibitor of AOB did not show an effective suppression to growth of AOB *amoA* genes, on the contrary, acted as a positive stimulate to the abundance of AOA *amoA* genes in the two test soils. Treatments with water or NO_3_- amendment which were applied with inhibitors or not did not change AOA *amoA* genes abundance significantly during the whole incubation (*P* > 0.05).

## Discussion

The abiotic processes and biotic processes including nitrification, denitrification are considered to be primary processes that produce N_2_O in arable soils from numerous reports^7,35,36^. This is first to distinguish the relative contribution of ammonia-oxidizing and N_2_O yield resulting from AOA and AOB in different pH of purple soil, southwest of China, using 1-octyne to specifically inhibit AOB. In this study, we verified that N_2_O emissions from the two tested agricultural soil were driven by nitrification and non-negligible others processes based on the following points: (1) NH_4_^+^-N rapidly transformed into NO_3_^–^N in the NH_4_^+^-N addition treatments and resulted in much higher N_2_O production than NO_3_^–^N addition treatments in which the NO_3_^–^N remained stable in during the incubation (Fig. 1); (2) both of test soils were performed under aerobic and 60% WFPS conditions which are optimal N_2_O production via ammonia oxidation according to previous studies6. And N_2_O emissions from heterotrophic denitrifiers were negligible^24^; (3) Nevertheless, N_2_O accumulated slowly but non-negligible in acetylene treated microcosms when NH_3_ oxidizer including AOA and AOB growth and activity were inhibited indicating that others processes–induced (heterotrophic nitrification and abiotic processes, etc.) N_2_O emission also hold an assignable contribution, especially in the neutral soils where others processes contribute 30.7% to the gross N_2_O emission in the incubation (Table S1 and Fig. 4).

Numerous studies have confirmed that N_2_O emissions in arable soils were regulated by multiple environmental factors such as soil moisture, soil pH and management factors including N application, plowing and other soil management treatments^37–39^. Soil pH is a key parameter that controls the abundance change of AOA and AOB which influence N_2_O production^40^. In our study, AOB contributed more accumulated N_2_O emissions than AOA in alkaline soil at the condition of NH_4_^+^-N addition which indicated AOB dominated the nitration process in alkaline soil, while there is no significant difference observe in neutral soil where both AOA and AOB contributed nearly a third of gross N_2_O emissions (Fig. 4 and Table S1). We found that AOB played a more important role than AOA in ammonia oxidation in a high-pH soil, supporting previous reports^21,22,41,42^. One possible explanation is that soils with higher pH accelerate the rate transformation of NH_4_^+^–N to availability NH_3_ which affected population and activity of ammonia oxidizers^27^, while bacterial growth would possibly have been impeded in low soil pH^43^.

Clearly, tested soils with NH_4_^+^ stimulated N_2_O production both by AOA and AOB to varying degrees with respect to the control (Fig. 2 and Table S1). And these results were also confirmed by the markedly increased *amoA* gene abundance of AOA and AOB at the end of incubation (Fig. 5). At present, the best explanation for different growth and activities of AOA and AOB in soils is a significantly different affinity for NH_3_^44–46^. For example, soils with high NH_4_^+^-N concentrate is conducive to the growth of AOB^16,20^, whereas AOA activity which is favored by low NH_4_^+^–N soil may be restricted or not affected in this condition of high NH_4_^+^–N ^47,48^. In our study, the AOA *amoA* abundance in the control of two test soil increased but not significantly, indicating that AOA could grow using organic N in low ammonia fertility status soils^22,49^. Unexpectedly, we found that AOA also grew in this condition where high NH_4_^+^-N concentration and AOB was inhibited by 1-octyne because the significant increase of AOA *amoA* abundance proved this point which was different from several previous studies (Fig. 5c, d)^21,28^. And this result was in line with a recent report that there was a direct competition between AOA and AOB for NH_3_ under high NH_4_^+^-N concentration when AOB was inhibited by 1-octyne, whereas AOA growth continued and AOB growth ceased when NH_4_^+^ -N became undetectable^24,50^.

When NH_4_^+^–N supplied as fertilizer, AOB dominated NH_3_ oxidation process and N_2_O yield ranged from 0.03 to 0.22%, which varied with soil type (Fig. 3). In neutral soil, the N_2_O yield induced by AOB (~ 0.03%) fell within the range values (0.02–0.09%) which were derived from soil-slurries with NH_4_^+^ amended experiment^51^. While in alkaline soil, the higher N_2_O yield of AOB (0.22%) which similar to the results of the pure culture of soil Nitrosospira lineage^52^ which predominate in soil AOB communities^53^. As for AOA, when addition of NH_4_^+^–N, N_2_O yield are 0.02% ~ 0.04% in the two test soils, and these yields are similar to values of 0.035% reported previously by Hink et al.^50^ and slightly lower than those of cultivated soil AOA (0.08% ~ 0.23%)^12,54,55^. These results suggested that AOB might have a higher N_2_O yield than AOA in the process of producing N_2_O in both alkaline and neutral soils. The higher N_2_O yield for AOB than AOA could be explained by the current acknowledgment: there were two enzymatic mechanisms for N_2_O production in AOB (i.e. nitrifier denitrification and incomplete NH_2_OH oxidation), while AOA appeared to lack a known NO reductase which was a key enzyme of reducing NO to N_2_O^56–58^. Therefore, N_2_O production of AOB was known as a biotic process, whereas inducing by AOA more liked a biotic and abiotic hybrid formation^59^.

In addition, the method of nitrification inhibitor addition has limitations on the effectiveness to distinguish relative N_2_O emission of AOA and AOB^21^. 1-octyne is an effective selective inhibitor of AOB activity and *amoA* abundance in test soils, and we found that AOB holds the leading role to soil N_2_O production in NH_4_^+^-N addition treatments although AOA *amoA* abundance showing several times of AOB in both of two soils (Figs. 4 and 5). Acetylene acted as a non-selective inhibitor which was used to block the activity of both AOA and AOB biotic ammonia oxidation, while the accumulated N_2_O production by others processes (heterotrophic nitrification and abiotic processes, etc.) contributed a remarkable scale to gross of accumulated N_2_O production (Fig. 4). Additional investigations like isotope labeling should be carried in the future to reveal the underlying mechanism of other processes leading to N_2_O.

At present, nitrification inhibitors^60^, slow-release fertilizers^61–63^, appropriate timing of fertilizer application^61,64^ and no-till^65,66^ were considered to be the main strategies to increase fertilizer use efficiency and inhibit N_2_O emission. Our results evaluated the consequences of specialization of ammonia oxidation accompanied by varied N_2_O yield of AOA and AOB which provide a potential strategy for the alleviation of N_2_O emissions in purple soils in hilly areas of upper Yangtze River, China. N_2_O accumulative production and N_2_O yield (especially in AOB) significantly increase with the rising pH of soils under aerobic conditions indicated a reduction in pH could be a potential way to decrease N_2_O emission. In addition, the application of a moderate nitrification inhibitor could both alleviate the N_2_O emission and reduce the risk of nitrate leaching in this area, and this viewpoint was supported by a recent review^67^. In all, measures that prevent ammonia oxidization directly or change specialization causing the increased dominance of NH_3_ oxidation by AOA indirectly will decrease the N_2_O production in this area. Meanwhile, crop yield, measures feasibility and cost should be considered with environmental due diligence which was derived from reducing N_2_O emission.

## Conclusions

In conclusion, we explored the relative contribution of ammonia-oxidizing bacteria and archaea to N_2_O emission using a selected inhibitor of AOB in purple soils with different pH values. Results demonstrated that ammonia oxidation was dominated by AOB rather than AOA under the 60% WHC soil moisture and aerobic condition in both neutral and alkaline soils. NH_4_^+^-N supply significantly increased N_2_O production of AOB, while AOA-related N_2_O production also increased when AOB activity was inhibited in this condition. pH act as a key factor to mediate the abundance change of AOA and AOB, and N_2_O production varied with different soils of pH. These results may help inform the development of N_2_O reduction strategies in the future.

## Supplementary Information

Supplementary Information 1.

Supplementary Information 2.

## Data Availability

All data generated or analysed during this study are included in this published article and its supplementary information files.
