# Supplementary material for: The contributions of ammonia oxidizing bacteria and archaea to nitrification-dependent N2O emission in alkaline and neutral purple soils
Source: Sci Rep. 2022 Nov 19;12:19928. doi: 10.1038/s41598-022-23084-1 (PMC9675842; doi:10.1038/s41598-022-23084-1)
Supplement: Supplementary file 2 — Supplementary Information 2. [file 41598_2022_23084_MOESM2_ESM.docx]

Table S1 Accumulated N_2_O emissions (mean ± standard error, μg N kg^−1^ dry soil) of two soils

| Treatment | SX | | PL | |
| --- | --- | --- | --- | --- |
|  | Day1 | Day21 | Day1 | Day21 |
| N-free | 0.42±0.00 d | 4.90±1.03 c | 0.34±0.04 d | 3.91±1.29 c |
| N-free + Ace | 0.39±0.02 d | 3.46±0.41 c | 0.22±0.02 de | 3.81±0.83 c |
| N-free + Oct | 0.45±0.05 d | 5.23±0.24 c | 0.24±0.00 de | 4.01±0.86 c |
| NH_4_^+^ | 7.01±0.61 a | 13.79±7.79 a | 32.88±6.38 a | 44.53±18.42 a |
| NH_4_^+^ + Ace | 0.72±0.04 c | 4.22±0.62 c | 0.62±0.05 c | 3.50±0.41 c |
| NH_4_^+^ + Oct | 1.91±0.23 b | 8.81±1.39 b | 3.81±0.63 b | 13.02±2.69 b |
| NO_3_^-^ | 0.32±0.02 d | 3.99±0.69 c | 0.22±0.03 de | 3.28±0.21 c |
| NO_3_^-^ + Ace | 0.47±0.03 d | 3.95±0.31 c | 0.13±0.01 e | 3.05±0.76 c |
| NO_3_^-^ + Oct | 0.49±0.03 d | 4.46±1.09 c | 0.25±0.02 de | 3.47±0.47 c |

Different letters in the same column indicate significant differences among treatments in two soils.
